# *In-vivo*^31^P-MRS of skeletal muscle and liver: A way for non-invasive assessment of their metabolism

**DOI:** 10.1016/j.ab.2017.01.018

**Published:** 2017-07-15

**Authors:** Ladislav Valkovič, Marek Chmelík, Martin Krššák

**Affiliations:** a High-field MR Centre, Department of Biomedical Imaging and Image-guided Therapy, Medical University of Vienna, Vienna, Austria; b Oxford Centre for Clinical Magnetic Resonance Research (OCMR), University of Oxford, Oxford, United Kingdom; c Department of Imaging Methods, Institute of Measurement Science, Slovak Academy of Sciences, Bratislava, Slovakia; d Christian Doppler Laboratory for Clinical Molecular MR Imaging, Vienna, Austria; e Institute for Clinical Molecular MRI in Musculoskeletal System, Karl Landsteiner Society, Vienna, Austria; f Division of Endocrinology and Metabolism, Department of Internal Medicine III, Medical University of Vienna, Vienna, Austria

**Keywords:** Phosphorus magnetic resonance spectroscopy, Energy metabolism, Skeletal muscle, Liver, Exercise-recovery, Saturation transfer

## Abstract

In addition to direct assessment of high energy phosphorus containing metabolite content within tissues, phosphorus magnetic resonance spectroscopy (^31^P-MRS) provides options to measure phospholipid metabolites and cellular pH, as well as the kinetics of chemical reactions of energy metabolism in vivo. Even though the great potential of ^31^P-MR was recognized over 30 years ago, modern MR systems, as well as new, dedicated hardware and measurement techniques provide further opportunities for research of human biochemistry. This paper presents a methodological overview of the ^31^P-MR techniques that can be used for basic, physiological, or clinical research of human skeletal muscle and liver in vivo. Practical issues of ^31^P-MRS experiments and examples of potential applications are also provided. As signal localization is essential for liver ^31^P-MRS and is important for dynamic muscle examinations as well, typical localization strategies for ^31^P-MR are also described.

## List of abbreviations

^1^H-MRS: proton magnetic resonance spectroscopy
^31^P-MRS: phosphorus magnetic resonance spectroscopy
^31^P-MRSI: phosphorus magnetic resonance spectroscopic imaging
ADP: adenosine-diphosphate
AMARES: advanced method for accurate, robust, and efficient spectral fitting
AMESING: adiabatic multi echo spectroscopic imaging
ATP: adenosine-triphosphate
BISTRO: B_1_-insensitive train to obliterate
CK: creatine-kinase
Cr: creatine
CSDE: chemical shift displacement error
CSI: chemical shift imaging
cST: continuous irradiation saturation transfer
DANTE: delays alternating with nutations for tailored excitation
DC: direct current
DRESS: depth-resolved surface coil MRS
FA: flip angle
FAST: four-angle saturation transfer
FID: free induction decay
G6P: glucose-6-phosphate
GPC: glycerol-phosphocholine
GPE: glycerol-phosphoethanolamine
INEPT: insensitive nuclei enhanced by polarization transfer
IR: inversion recovery
ISIS: image-selected in vivo spectroscopy
IT: inversion transfer
MT: magnetization transfer
MVC: maximal voluntary capacity
NAD (NAD^+^, NADH): nicotinamide adenine dinucleotide (its oxidized and reduced form, respectively)
NAFLD: non-alcoholic fatty liver disease
NASH: non-alcoholic steatohepatitis
NET: non-equilibrium thermodynamic
NOE: nuclear Overhauser effect
OVS: outer-volume saturation
PAD: peripheral arterial disease
PC: phosphocholine
PCr: phosphocreatine
PDE: phosphodiesters
PE: phosphoethanolamine
Pi (Pi_2_): inorganic phosphate (alkaline/mitochondrial Pi)
prST: progressive saturation transfer
pST: pulsed saturation transfer
PtdC: phosphatidylcholine
RF: radio-frequency
semi-LASER: slice-selective excitation with localization by adiabatic selective refocusing
SNR: signal-to-noise ratio
ST: saturation transfer
STEAM: stimulated echo acquisition mode
SVS: single-voxel spectroscopy
tCr: total creatine
TE: echo time
TR: repetition time
TRiST: triple repetition time saturation transfer
TwiST: two-repetition time saturation transfer
UDPG: uridine diphosphate glucose
VOI: volume of interest

## 1 Introduction

Phosphorus magnetic resonance spectroscopy (^31^P-MRS) and spectroscopic imaging (^31^P-MRSI) offer unique, non-invasive windows into the metabolism of human tissues [1], [2], [3]. In addition to the information contained within the static spectra, ^31^P-MRS also provides techniques for the assessment of the rates of chemical reactions that are involved in energy metabolism [4], [5]. Of particular interest is also the possibility to investigate the oxidative energy production by mitochondria in skeletal muscle during exercise and subsequent recovery [6], [7]. The current progress in MR hardware, represented by the ultra-high magnetic field strength of in vivo MR systems and improved dedicated radio-frequency (RF)-coil technology, provides significant benefits for ^31^P-MRS, and therefore, attracts many scientists to explore its potential.

**Fig. 1:**
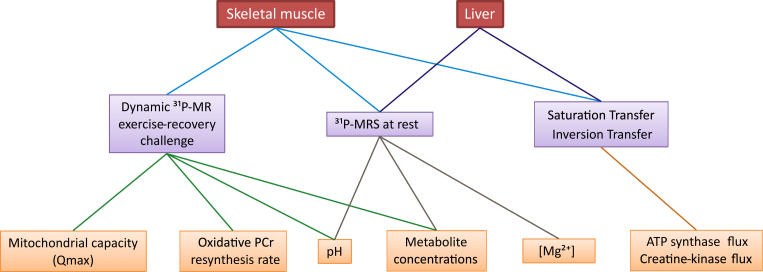
An overview of the biochemical parameters (orange) assessed and quantified through in vivo ^31^P-MRS/MRSI experimental approaches reviewed and discussed in the text (violet) in the organs of interest, i.e. skeletal muscle and liver, (red).

This review provides an overview of the current status of in vivo ^31^P-MRS with a focus on the techniques for energy metabolism measurement applied to human skeletal muscle and liver. Aspects of static spectra acquisition, dynamic muscle experiments, and saturation transfer (ST) methods are discussed. Fig. 1 depicts the covered topics and their associations.

### 1.1 Information content of the ^31^P-MR spectrum

**Fig. 2:**
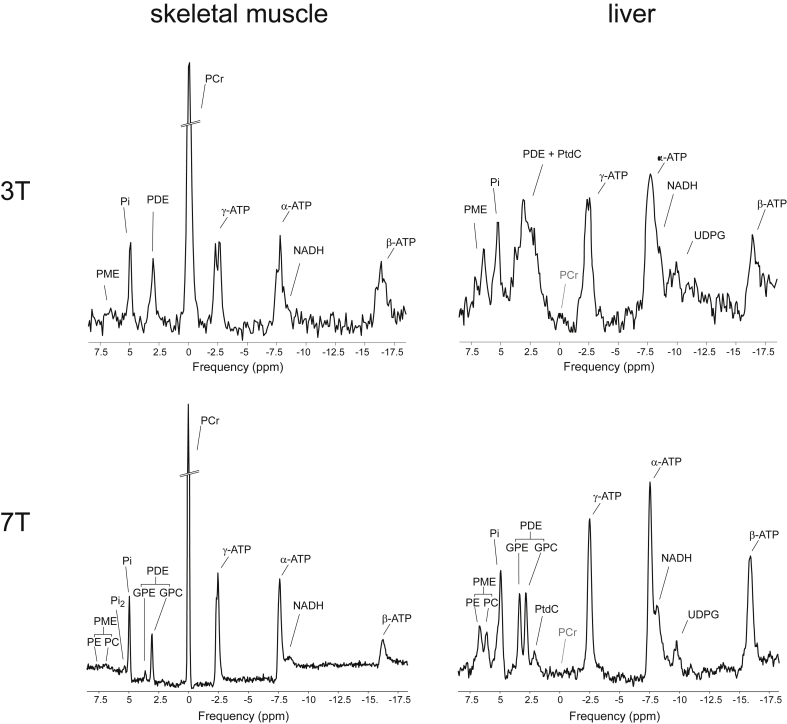
Typical ^31^P-MR spectra acquired at 3 T (top) and 7 T (bottom), at rest, in skeletal muscle (left) and liver tissue (right). All spectra are depicted relative to the resonance frequency of phosphocreatine (PCr), although this is not present in healthy human liver tissue. Phosphorus metabolites common to both tissues include resonance lines of adenosine-triphosphate (ATP), nicotinamide adenine dinucleotide (NADH), phosphodiesters (PDEs) – glycerol-phoshocholine (GPC) and glycerol-phosphoethanolamine (GPE), inorganic phosphate (Pi) and phosphomonoesters (PMEs) – phosphocholine (PC) and phopshoethanolamine (PE). Note that PDEs and PMEs are readily resolved at 7 T. Another metabolite resolved in the muscle at 7 T is the recently described alkaline Pi (Pi_2_) pool. The liver spectrum, on the other hand, contains a resonance line of uridine diphosphate glucose (UDPG) and also a recently assigned spectral line of the bile component phosphatidylcholine (PtdC). Note that, due to the large frequency range at 7 T and the in vivo linewidth, the J-coupling of the ATP resonances is no longer resolved and the frequency limitations of the excitation pulse cause the β-ATP frequency line to be suppressed.

The dominant metabolite signal in the ^31^P-MR spectrum is singlet of the phosphorylated form of creatine (Cr), i.e. phosphocreatine (PCr). The high energy phosphate bond in PCr serves as a rapidly available energy reserve, ready to replenish energy catalyzed from adenosine-triphosphate (ATP) during increased energy expenditure. ATP contains three non-equivalent phosphate groups (α-, β-, and γ-) that differ in their resonance frequencies, which yield three individual signals in the ^31^P-MR spectra. As the phosphate nuclei in ATP interact with other nearby spins, i.e. undergo so-called homonuclear J-coupling, line splitting of the ATP signals can be observed, which forms doublets for α- and γ-ATP, and a triplet for β-ATP. Another important metabolite visible in the ^31^P-MRS spectrum is inorganic phosphate (Pi), which also serves as a substrate or product in chemical reactions of energy metabolism. Fig. 2 (left) depicts typical in vivo ^31^P-MR spectra from human skeletal muscle and Fig. 2 (right) depicts typical ^31^P-MR spectra from human liver. The main difference between these two spectra is obvious: there is no PCr signal in healthy liver.

Other detectable ^31^P metabolites include cell membrane precursors, i.e. phosphomonoesters (PMEs) and cell membrane degradation products, i.e. phosphodiesters (PDEs). The major contributors to the PME signal in vivo are phosphocholine (PC) and phosphoethanolamine (PE), while the main PDEs are glycerol-phosphocholine (GPC) and glycerol-phosphoethanolamine (GPE). In skeletal muscle, however, the contribution of GPE is only minimal and the main PDE signal is, therefore, GPC (Fig. 2a). Recently, an additional phospholipid signal, i.e. phosphatidylcholine (PtdC), was recognized in the human liver spectra, which presumably originates from bile, and its assessment could potentially hold diagnostic value in bile duct and liver disorders [8]. Nicotinamide adenine dinucleotide (NAD) in its oxidized and reduced form (NAD^+^ and NADH, respectively) and uridine diphosphate glucose (UDPG) can be also detected in ^31^P-MR spectra, particularly when using ultra-high fields, such as 7 T.

The amount of visible phosphorus-containing metabolites constitutes important, but not the only information that can be gained from the analysis of ^31^P-MR spectra. The concentration of adenosine-diphosphate (ADP) is under physiological conditions too low to be detectable by in vivo MRS. However, as will be shown later, ADP can be indirectly calculated using information about PCr, ATP, and Cr, due to its involvement in the creatine-kinase (CK) reaction. Equally, the detection of glucose-6-phosphate (G6P) in skeletal muscles in vivo is severely hampered by its low tissue concentration under normoglycemic-normoinsulinemic conditions and its spectral overlap with other PME signals. However, increase in G6P levels during either euglycemic- or hyperglycemic-hyperinsulinemic clamp can be quantified by subtracting the initial resting spectra [9], [10], [11]. The chemical environment of phosphate nuclei in compounds, which defines their resonance positions in ^31^P MR spectra, may change with physiological or pathological conditions. These variations can be, in turn, used to indirectly define the actual physiological conditions under which the spectrum was acquired. Of particular importance is the effect of pH on the exact spectral resonance position of inorganic phosphate (Pi) [12]. While the position of the PCr peak remains constant, Pi resonance changes with pH. Thus, the chemical shift difference between PCr and Pi (δ) can be used to derive pH values using the modified Henderson-Hasselbach equation:

(1) pH=pKA+log[(δ−δHA)(δA−δ)]

where pK_A_ = 6.75 is the dissociation constant of Pi, δ_HA_ = 3.27 is the chemical shift of the protonated form of Pi, and δ_A_ = 5.63 is the chemical shift of the non-protonated form of Pi. As the dominant signal for Pi in the skeletal muscle spectra arises from sarcoplasm, it is the intramyocellular pH that can be determined in this way [7]. Another physiological condition that may influence the resonance positions of ^31^P metabolites is the cellular content of free Mg^2+^, which plays an important role in diverse intracellular biochemical reactions. In particular, the Mg^2+^ complexes of ADP and ATP act as substrates for ATPases and kinases. The amount of free Mg^2+^ can be calculated from the chemical shift between β-ATP and α-ATP [13] or from the chemical shift between β-ATP and PCr [14].

### 1.2 Particular differences and technical requirements compared to proton (^1^H)-MRS

^31^P-MRS cannot compete with the abundant ^1^H-MRS in signal sensitivity; however, it has other advantages. Next to its already mentioned ability to measure pH or quantify ADP and Mg^2+^ concentrations, ^31^P-MRS disposes a significantly larger spectral width, leading to better separation of metabolites. In particular, PC, PE, and GPC that are readily separated in ^31^P-MR spectra are all choline-containing compounds that cannot be differentiated by ^1^H-MRS in vivo. There is also no dominant water or fat signal present in the ^31^P-MR spectra, and thus, no frequency-selective suppression techniques are necessary. Another difference is the spin lattice relaxation dependence on the external magnetic field. While, for ^1^H-MRS, the leading relaxation mechanism is the magnetic dipole-dipole interaction, the relaxation of ^31^P metabolites is strongly influenced by a competing mechanism called chemical shift anisotropy, which prevails at higher field strengths [15], [16].

The main difference lies, however, in its Larmour frequency (ω_0_). As all clinical MR systems focus on the use of ^1^H nuclei for clinical imaging, they are tuned for the ω_0_ of ^1^H. Thus, to be able to use these systems for ^31^P-MR, additional hardware, i.e. a broadband transmitter and a receiver, as well as RF-coils, is necessary. To compensate for the low sensitivity of MR systems to ^31^P, surface coils that offer very high sensitivity in their close vicinity are often applied for organ-specific ^31^P-MR investigations [17], [18], [19], [20]. These are commonly constructed as simple single loops; however, several complex coil-arrays that provide higher sensitivity and better volume coverage have been introduced recently [21], [22], [23], [24]. While the receive sensitivity of such RF-probes is undoubtedly superior, their transmit efficiency, particularly at ultra-high fields, may be insufficient [23], [25]. The inhomogeneous excitation profiles of these RF-coils introduce strong flip-angle (FA) variability, which can significantly influence the quantification of ^31^P-MRSI data. To be able to correct for this spatial distribution, exact knowledge of the FA map is required. Techniques reported recently have been developed especially for FA mapping of ^31^P RF-coils at high fields [26], [27], where simulations and phantom replacement techniques might become unreliable.

The combination of the receive sensitivity of modern coil-arrays with the transmit power, B_1_ homogeneity, and volume coverage of volume coils, e.g., the recently proposed volume coil for 7 T [28], could, in the future, constitute a prime tool for ^31^P-MR at ultra-high fields.

### 1.3 How to improve the quality of ^31^P-MR spectra

Undoubtedly, similar to ^1^H-MRS, high homogeneity of the static magnetic field B_0_ increases the quality of the ^31^P-MR spectra. Still, there are also other options for further improving the spectral resolution and/or signal-to-noise ratio (SNR) of the ^31^P-MRS data.

Analogously to the already mentioned homonuclear spin coupling in ATP, there are also heteronuclear interactions between phosphate and proton spins in many other phosphorus-containing metabolites. This ^1^H—^31^P heteronuclear coupling broadens the resonance lines, particularly of PDEs and PMEs compounds, in the in vivo spectra. Applying RF irradiation at the ^1^H frequency during ^31^P signal acquisition can effectively decouple these interactions, yielding narrower and higher spectral lines [29]. Broadband ^1^H decoupling allows for separate quantification of metabolites that contribute to overall PDE and PME signals, which otherwise cannot be separated at lower fields (B_0_ ≤ 3 T), i.e. GPC and GPE, as well as PC and PE. As nothing is free in MRS, this increase in spectral resolution comes at the cost of increased power deposition, i.e. specific absorption rate (SAR), due to the long, intense RF-pulses used for ^1^H decoupling.

Another option for signal enhancement is the utilization of the nuclear Overhauser effect (NOE) to transfer magnetization from ^1^H to the ^31^P nuclei. Using the NOE, through ^1^H RF-irradiation during the ^31^P inter-pulse delay, the detection sensitivity for ^31^P can be increased up to about 80% at lower fields in skeletal muscle [30]. The reported increase of up to 44% in brain [31] and prostate [32] in ^31^P-MR signal at ultra-high fields cannot be directly transferred to skeletal muscle or liver, due to the NOE dependence on the tissue of interest. It is also important to note that the effect is metabolite-specific, and therefore, individual correction factors must be applied for reliable quantification. Similar to ^1^H decoupling, the NOE leads to significant power deposition, increasing SAR and limiting its use at ultra-high fields.

A further technique for the enhancement of ^31^P sensitivity is the insensitive nuclei enhanced by polarization transfer (INEPT) [33]. Polarization of the excited ^1^H spins is transferred through heteronuclear spin coupling to the ^31^P spins, e.g., in PMEs and PDEs. This happens during the period TE_1H_ period, which has to be relatively long for optimal effect, thus, causing counteracting signal loss due to T_2_ relaxation. This can be partially compensated by the use of short TRs, utilizing the large T_1_ differences between ^31^P and ^1^H spins [34]. Similarly, adiabatic multi echo spectroscopic imaging (AMESING [35]) could be used to regain the sensitivity lost due to T_2_^∗^ losses [36].

A very important improvement in ^31^P-MR spectral quality, in terms of more than double the SNR and spectral resolution, comes through the use of ultra-high field MR systems (B_0_ ≥ 7 T) [15], [16], [18], [31], [37], [38]. Additional SNR per unit of time can be gained due to the shortening of the T_1_ relaxation times of phosphorus metabolites at 7 T [15], [38]. This is due to the previously mentioned chemical shift anisotropy dominance over the relaxation of ^31^P metabolites at 7 T [15]. The increase in SNR at ultra-high fields can be, in turn, exchanged for higher spatial or temporal resolution for time-demanding experiments, such as ST [39]. Therefore, ultra-high field MR systems (B_0_ ≥ 7 T) hold great potential for investigations of the not yet well-understood mechanisms of human energy metabolism.

### 1.4 Spectral processing

Next to the acquisition of high-quality data, the application of an appropriate processing technique is required to obtain reliable results. Prior to the actual quantification, the acquired data have to be pre-processed. The main ^31^P-MR spectra pre-processing step is the zero-order phase and the first-order phase correction. Data filtering is typically avoided during pre-processing and is used only for spectra visualization. A special case is a filter that does not cause any line broadening, e.g., a so-called line-width matched filter, which is occasionally applied in case of very-low SNR data [40]. Zero filling is also used to pre-process individual ^31^P-MR spectra. It is more commonly used in MRSI acquisition to zero-fill in the k-space during matrix interpolation.

A frequent quantification approach constitutes the application of a time domain fitting algorithm, which incorporates prior knowledge for improved spectral fitting. In particular, the advanced method for accurate, robust, and efficient spectral fitting (AMARES) routine [41] offers high flexibility of the fitting parameters and is, therefore, most commonly used for the analysis of ^31^P-MR spectra. AMARES is freely available as a part of the jMRUI software package [42], or as a MATLAB (Mathworks Inc, Natick, MA) function [43]. The possibility to include prior knowledge, e.g., resonance frequency and/or linewidth, and thus constrain the fitting parameters, can significantly improve/influence fitting performance. It is, therefore, important to clearly state any restrictive prior knowledge when reporting study results.

#### 1.4.1 Quantification – relative vs. absolute

In addition to quality measures, e.g., SNR, linewidth of the peak (reflecting the quality of the shim), and the Cramer-Rao Lower Bound (quality of the spectral fitting), the results of spectral analysis include information about the first point of the free induction decay (FID), the amplitude of which is directly proportional to the area (integral) of the metabolite peak. It is common practice to publish the results of in vivo MRS, especially in the clinical field, as metabolite ratios. However, metabolite ratios cannot provide unambiguous information about metabolic changes, as encountered in many disorders and pathology studies with in vivo MRS. In order to achieve reliable statements about metabolic changes, it is crucial that absolute concentrations are obtained.

MRS can, in principle, be used to assess absolute concentrations in mmol L^−1^ or μmol g^−1^ of tissue in vivo. It is based on the fact that the thermal equilibrium magnetization, M_0_, is directly proportional to the number of spins (n), which is proportional to the molar concentration, and is given by the equation:

(2) M0=(γ.h2.π)(n.B04.k.T)

where γ is gyromagnetic ratio, h is the Planck constant, k is the Boltzmann constant, and T is absolute temperature.

In an MRS experiment, the acquired metabolite signal, S_met_, is not directly M_0_, but depends on many experiment-specific factors:

(3) $${\text{S}}_{\text{met}}=\text{NA}\text{.RG}.{\text{ω}}_{0}.\left[{\text{c}}_{\text{met}}\right].\text{V}.{\text{f}}_{\text{seq}}.{\text{f}}_{\text{coil}}$$

where NA is the number of averages, RG is the receiver gain, [c_met_] is the molar concentration of the metabolite, V is the volume size, and f_seq_ and f_coil_ are functions that describe the signal modulations due to the pulse sequence and RF coil used, respectively. f_seq_ depends on the repetition time (TR), the echo-time (TE), the number and type of RF pulses and on the T_1_ and T_2_ relaxation times. f_coil_ accounts for factors related to the quality and the geometry of the RF-coil (e.g., the quality factor Q and filling factor). Direct calculation of the metabolite concentration [c_met_] from the detected signal, S_met_, is not possible, because some of the coil-related factors in the f_coil_ function are unknown. Therefore, all quantification methods utilize a calibration or reference compound of known concentration [c_ref_] to which the metabolite signals are referenced. The metabolite concentration can be then calculated according to:

(4) [cmet]=[cref].SmetSref.CMR

where S_ref_ is the signal detected from the reference compound and C_MR_ is a correction factor accounting for differences in relaxation times, γ, diffusion, magnetic susceptibility, spatial position relative to the coil, and, in general, any other differences between the reference compound and the metabolite.

Generally, there are three approaches to convert relative numbers to absolute concentrations [44], [45], [46]. The first approach uses an internal concentration reference, which can be established from a stable metabolite that occurs naturally in the tissue. The second approach utilizes an external concentration reference positioned outside the object under investigation, but within the sensitive area of the coil. The third approach also uses an external concentration reference, which is, however, measured in a separate experiment. This is known as the phantom replacement technique.

A quantitative review of ^31^P MRS absolute quantification in muscle, published recently [47], concluded that, in healthy human muscle, metabolite concentrations can be safely obtained from uncalibrated ^31^P MRS measurements using muscular ATP concentration, which is assumed to be stable ([ATP] ≈ 5.5 mmol/kg wet weight ≈ 8.2 mmol/L cell water [48], [49], [50], [51]), as an internal concentration reference. Whether this is also the case in muscle affected by disease remains to be shown in further studies.

For quantification of the molar concentration of the ^31^P metabolites in human liver, it is essential to avoid skeletal muscle contamination. Therefore, it is very common to use techniques to select a volume in this organ [52], [53], [54], [55], [56], [57], [58], [59], [60], including the suppression of signal from abdominal muscle. Investigations on MR systems at 1.5 T reported highly discrepant hepatic metabolite concentrations, which was later investigated and attributed to T_1_ dependent saturation losses, as well as to differences in post-processing and quantification methods [59]. Recent studies with improved data quality at 3 T resulted in better reproducibility in a clinically acceptable time of around 30min [53], [54].

## 2 ^31^P-MRS of skeletal muscle

Because of its high metabolic activity, physiological importance, and relatively simple access, skeletal muscle of the lower or upper leg was the first human tissue studied by ^31^P-MRS in vivo [2], [61], [62]. These experiments confirmed the results of animal studies, which suggested that ^31^P-MRS can probe the human energy metabolism non-invasively [1] and encouraged numerous subsequent investigations. Next to the analysis of resting ^31^P-MR spectra, of particular interest is the possibility of obtaining the ^31^P-MR spectra in a dynamic fashion, with sufficient time resolution during an exercise challenge and consecutive recovery [2], [63]. The option to measure the dynamics of important chemical reactions in vivo through a magnetization transfer technique further increases the impact of ^31^P-MRS. All of these techniques are described in more detail in the next paragraphs, and although a detailed justification of the clinical implications of ^31^P-MRS is out of the scope of this methodological review, a few examples of its applications are provided to demonstrate its potential.

### 2.1 ^31^P-MRS of resting muscle

The most straightforward application of ^31^P-MRS is the acquisition of spectral transients at rest. The exact number of the transients depends on the parameters of the sequence used and on the required SNR. SNR enhancement techniques, e.g., NOE or ^1^H decoupling, described above, can be also applied, but have implications for the quantification of metabolites. The quantification (either relative or absolute) of such static ^31^P-MR spectra can be used to gather information about skeletal muscle fiber composition or the assessment of training status/fitness. Changes in relative ^31^P metabolite concentrations, i.e. a drop in PCr and an increase in Pi, were also observed in patients with mitochondrial myopathy [64], [65]. Increased levels of PDE measured at rest can be indicative of congenital lipodystrophy [66], fibromyalgia [67], or muscular dystrophies [68], [69]. It is also worth mentioning that changes in PCr/Pi or total (PCr+Pi) levels after exercise were attributed to muscle damage caused by a strenuous lengthening exercise [70]. And, as such damage may persist for two weeks, static ^31^P-MRS might potentially offer a tool for the non-invasive monitoring of muscle fitness during recuperation.

#### 2.1.1 Muscle fiber composition

Skeletal muscles consist of a large number of muscle fibers. These can differ in their contractile (slow-twitch vs. fast-twitch) and metabolic (oxidative vs. glycolytic) properties. In humans, three main fiber groups have been defined [71]. Type I or slow-twitch oxidative fibers heavily rely on the oxidative capacity of the mitochondria and a high triglycerides reserve. Type IIb or fast-twitch glycolytic fibers demonstrate high ATPase activity and high glycolytic capacity, and Type IIa fast-twitch oxidative glycolytic fibers with mixed high oxidative and glycolytic activities [72]. The proportion of the fiber types within the muscle can be influenced by training [73], [74] and defines the main metabolic activity and influences the performance of the muscle in short and long challenges [75]. Although a muscle biopsy can provide information about fiber-type composition, the limited sample size and its invasive nature hinders large-scale use, particularly in repetitive studies.

Experiments performed on lower mammals, i.e. mice, rats and cats, showed that the muscle fibers differ in their content of energy-rich phosphates [76], [77]; therefore, ^31^P-MRS has been suggested as an alternative, non-invasive approach to muscle biopsy for the determination of fiber-type composition in human muscles. And, although several human studies have also found significant, but much smaller, differences in basal concentrations of PCr and/or Pi (or their ratio) between muscles containing mainly slow-twitch or fast twitch fibers, the scattering in metabolite content observed is large and the final conclusions vary [78], [79], [80], [81], [82], [83], [84], [85], [86], [87], [88]. This inconsistency complicates the straightforward use of resting ^31^P-MRS for muscle fiber composition determination, and potentially suggests that the basal phosphate content in humans does not correlate well with the defined muscle fiber classification [84].

#### 2.1.2 ^31^P-MRS at rest and training status

^31^P-MRS of skeletal muscle at rest has been extensively used to assess training status. Fiber-type composition was suggested as a potential marker of muscles fitness, as sedentary subjects rely mostly on fast-twitch Type IIb fibers, but, these studies, again, led to contradictory results [83], [84], [89].

A different approach toward the characterization of training status from static spectra is the use of the concentration of the cell membrane phospholipids - phosphodiesters [50], [83], [90], [91], [92]. At ultra-high fields (i.e., 7 T), or by using ^1^H decoupling, the main phosphodiester in human skeletal muscle – GPC – can be separately evaluated and used directly rather than the total PDE signal [92]. A sedentary lifestyle, in particular, if accompanied by overweight, gives rise to significantly higher PDE levels [92]. This difference cannot be explained through the relation of PDE to body mass index [50], [92] alone, as the PDE levels were also shown to relate directly to energy metabolism of skeletal muscle, measured by dynamic or transfer-related ^31^P-MRS techniques [50], [92]. Increased PDE levels, although to a much lesser extent, have been also reported in professional cyclists [90], [91] in comparison to normally trained students. Long-distance runners also exhibit higher PDE levels than sprinters [83]. Of note is the age dependence of the muscular PDE content. As the PDE level tends to increase with age [50], [92], [93], special care has to be taken for age-matching of study participants, when PDE content is to be used as a marker of muscle fitness. Nevertheless, the concentration of muscle PDE (or GPC) measured at rest provides valuable information about training status.

**Fig. 3:**
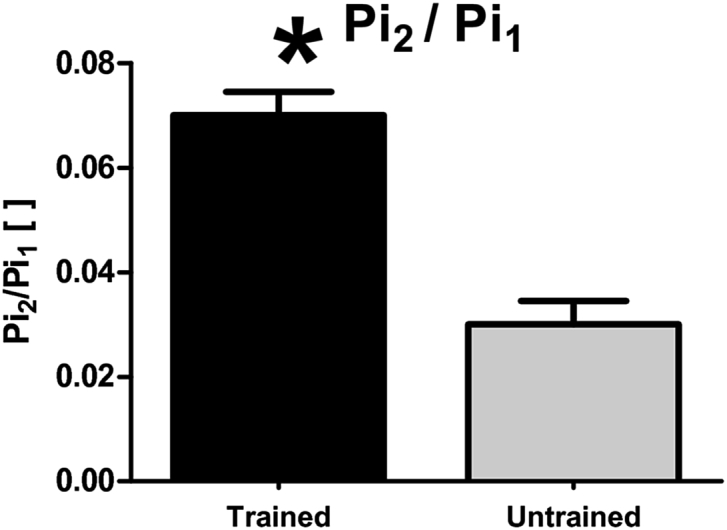
Bar plot showing a significantly higher Pi_2_/Pi (marked here as Pi_2_/Pi_1_) in the endurance trained athletes compared to the normal physical active group. Similarly, the Pi_2_/Pi was found lower in overweight-to-obese sedentary subjects than in lean, active individuals [92]. Figure was reproduced from Ref. [95].

A very recent approach for the determination of training status from resting ^31^P-MR spectra profits from the increased spectral resolution of the ultra-high field systems (i.e., 7 T), where an alkaline pool of Pi signal (Pi_2_) can be identified [94]. Based on its chemical shift (∼5.1 ppm), relatively short T_1_, and small contribution of extracellular space to skeletal muscle signal, the mitochondrial matrix has been recognized as the likely origin of this pool [94]. As such, it should be able to provide direct information about changes in mitochondrial density in response to training or defects of mitochondrial metabolism. A comparative study [95] showed an increased Pi_2_/Pi ratio in the quadriceps of the trained subjects (Fig. 3), and, thus, supported this hypothesis. Significantly lower Pi_2_ concentrations and Pi_2_/Pi ratios have also been reported in the overweight-to-obese sedentary subjects when compared to a group of lean, active individuals [92].

### 2.2 Dynamic ^31^P-MRS during exercise-recovery challenge

^31^P-MRS measurement of the kinetics of intramyocellular pH and of the cytosolic concentrations of PCr, Pi, and ADP during perturbations of metabolic equilibrium can be generalized as dynamic ^31^P-MRS. The disturbance in metabolic balance is usually achieved through muscle contraction and recovery. Unlike in static investigations, a low number of transients (commonly just one) is used in dynamic examinations due to the high temporal resolution required (on the order of seconds). Through the measurement of post-exercise recovery, dynamic ^31^P-MRS allows direct investigation of the pH homeostasis, as well as of the oxidative ATP synthesis regulation in relation to ATP demand [6], [63], [96], [97]. Therefore, if technically possible, dynamic ^31^P-MRS is the method of choice for the investigation of the mitochondrial metabolism of skeletal muscle in vivo.

Examinations of the oxidative metabolism of the skeletal muscles provide not only important information about muscle physiology [3], but can also be used to observe the effects of aging [98], [99] and/or to help define the training status [51], [92]. In addition, dynamic ^31^P-MR examinations can identify mitochondrial defects in muscular diseases, such as Duchenne's muscular dystrophy [100] or mitochondrial myopathy [101]. Furthermore, dynamic ^31^P-MRS could uncover decreased oxidative metabolism of skeletal muscle in patients with diabetes mellitus [102], [103], [104], heart failure [105], or peripheral arterial disease [106], [107], [108].

#### 2.2.1 Underlying physiology

A detailed description of the underlying physiology is out of the scope of this review and has been recently reviewed elsewhere [6]. Here, we provide only a brief introduction that is essential for an understanding of the role of dynamic ^31^P-MRS in examinations of skeletal muscle oxidative metabolism, along with the necessary equations for its basic evaluation.

The immediate source of energy for muscle contractions is the hydrolysis of ATP into ADP and Pi:

(5) $$\text{ATP}+{\text{H}}_{2}\text{O}\to \text{ADP}+\text{Pi}+{\text{H}}^{+}$$

During exercise, this reaction is driven mainly by the force-generating role of the myosin ATPase enzyme and also partially by other ATPases involved in Ca^2+^ homeostasis. The ATP pool within the muscle tissue is limited, and therefore, would only last for a brief period of contractile activity. ATP is in skeletal muscle produced either by glycolytic conversion of glucose to lactate (anaerobic respiration) or by oxidative phosphorylation (aerobic respiration). The latter is preceded by entering carbons from the glycolytic pathway via acetyl coenzyme A into the TCA cycle, and subsequent oxidation of NADH in the mitochondria. As both of these pathways require some time to start, any temporary mismatch between ATP demand and supply is compensated by immediate consumption of energy reserves stored in PCr.

(6) $$\text{PCr}+\text{ADP}+{\text{H}}^{+}↔\text{ATP}+\text{Cr}$$

**Fig. 4:**
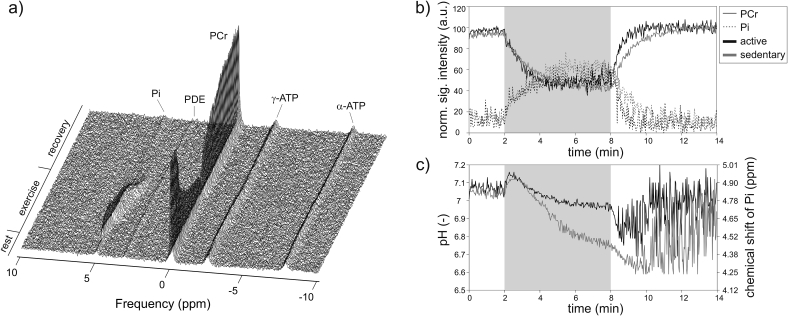
The ^31^P-MRS dynamic experiment with an isotonic aerobic exercise at a single workload (25% of maximal voluntary contraction force). A stack of dynamically acquired spectra is depicted in a). Note that while PCr depletes and Pi rises, ATP levels remain constant. The time-courses of the normalized PCr (full line) and Pi (dotted line) signal intensities are given in b). Panel c) shows the dynamic evolution of the calculated pH based on the chemical shift of Pi. In order to demonstrate the differences in training status observable by dynamic ^31^P-MRS, data from a regularly active (black lines) and sedentary (grey lines) volunteer are visualized in panels b) and c). The grey area indicates the 6-min long exercise period.

This temporal buffering reaction, catalyzed by the creatine-kinase (CK) enzyme, ensures that ATP concentration is virtually constant [109]. The consequential depletion of PCr is matched by the growth of free creatine (Cr), such that the total Cr (tCr) level remains stable (tCr = PCr + Cr). Similarly, the level of Pi also rises, such that the sum of PCr and Pi remains approximately constant. As illustrated in a stack plot of spectra acquired during dynamic examination in Fig. 4a, and the normalized time courses of the PCr and Pi signals in Fig. 4b, ^31^P-MRS provides a direct evidence of these processes.

The CK-driven PCr hydrolysis is also one of the processes involved in the stabilization of cellular pH. However, its rapid onset typically results in a small initial rise in pH (alkalization) after the start of the exercise (Fig. 4c). The fall in PCr and/or rise in pH also leads to an increase in free ADP, which still is much lower than ATP and bellow the ^31^P-MRS sensitivity in vivo. However, the concentration of ADP can be calculated according to CK equilibrium expression [110], which follows solution thermodynamics in skeletal muscle [111], using the equilibrium constant K_CK_ ≈ 1.66 × 10^9^ M^−1^ [112]:

(7) [ADP]=[Cr].[ATP][PCr].[H+].KCK

To solve equation (7), the concentration of free Cr must be estimated. There are two commonly used approaches for this quantification. One is based on the assumption that, under resting conditions, PCr represents ≈85% of tCr [96]. The other assumes a constant concentration of [tCr] ≈ 42 mM within the muscle tissue [47], [49].

The resynthesis of PCr after exercise can be considered entirely aerobic; therefore, the absolute PCr resynthesis rate measures the suprabasal ATP synthesis rate [6]. The time constant of PCr recovery (τ_PCr_), or the PCr recovery rate constant (k_PCr_ = 1/τ_PCr_), can be fitted by a monoexponential function [109], given by:

(8) [PCr](t)=[PCr]endexercise+Δ[PCr].(1−exp(−tτPCr))

where [PCr]_end_exercise_ is the concentration of PCr at the end of exercise; and ΔPCr is the difference between [PCr] at rest and [PCr]_end_exercise._ A higher order exponential function can be also used for τ_PCr_ fitting, if appropriate. The slow component of PCr recovery would then be related to the interactions between pH and PCr in the CK equilibrium [113]. The initial rate of PCr resynthesis (V_iPCr_) during the recovery period, which roughly represents the oxidative phosphorylation flux [114], can be calculated as follows:

(9) ViPCr=ΔPCrτPCr

To quantify the maximal rate of oxidative ATP synthesis from the PCr recovery data (Qmax), i.e. mitochondrial capacity, one of three basic ‘models’ is commonly used.

The first assumes a linear relationship between the PCr depletion and the initial recovery rate, where the slope of the relationship is defined by the exponential recovery rate constant, k_PCr_. Thus, the linear model often uses k_PCr_ (or τ_PCr_) as a relative inverse measure of metabolic function. This can be extended by theoretically extrapolating the V_iPCr_ for maximal PCr depletion, i.e. the maximal ATP synthesis rate [51], [115]:

(10) Qmax-lin=[PCr]restτPCr

The linear model offers only a rough estimation of the oxidative ATP synthesis rate, as both τ_PCr_ and Q_max-lin_ are highly dependent on the pH at the end of exercise [116], and is, therefore, valid only when pH changes are small. Besides, the calculation of Q_max-lin_ does not account for the resting rate of ATP turnover.

An alternative, more accurate model is based on the Michaelis-Menten dependence of oxidative metabolism on [ADP], i.e. the ‘ADP-based model’ [117]. The concentration of ADP at the end of exercise ([ADP]_end_exercise_), which can be calculated from equation (7), provides a direct relation to mitochondrial capacity (Q_max-ADP_). The simple, commonly used, first-order, hyperbolic approximation given in equation (11) does not account for the whole dynamic range, without unrealistic assumptions about the basal ATP turnover rate.

(11) Qmax-ADP=ViPCr+(1+Km[ADP]end_exercise)

Therefore, a sigmoid relationship with a Hill coefficient (n_H_ ≈ 2), which also includes basal rate of ATP turnover (Q_b_ ≈ 1.5–2.4 mM/min [114], [118]), was defined to provide a better fit [6],:

(12) Qmax-ADP=(ViPCr+Qb){1+(Km[ADP]end_exercise)nH}

K_m_ is the value of [ADP] at the half-maximal oxidation rate, which is approximately 30 μM in skeletal muscle tissue [63]; however, values in the literature range between 22 μM [114] and 44 μM [118]. To avoid the assumptions on fixed K_m_, Q_b_, and n_H_ values, Q_max-ADP_ can be calculated from a revised equation (12), and converted into a multi-point sigmoidal fitting function [119]:

(13) VPCr(t)=Qmax-ADP{[ADP](t)(Km+[ADP](t))}nH−Qb

where V_PCr_(t) is the oxidative resynthesis rate of PCr during recovery at a certain time point, which can be calculated from the derivative of the fitted PCr recovery time course in equation (8).

The third model is based on the non-equilibrium thermodynamic (NET) approaches, i.e. the ‘NET mode. l’ The simplest case uses the relationship between oxidative ATP synthesis and the free energy of ATP hydrolysis (ΔG_ATP_), which is supposedly quasi-linear [109]. Then, the rate constants of PCr or ΔG_ATP_ can be used as relative measures of metabolic function [6]. However, the linear relationship is valid only in a small range of ΔG_ATP_ values, being more exactly sigmoid [114], [118], [120], [121]. More complex fits have be used to obtain an extrapolated NET-model maximum flux [122]:

(14) Qmax-NET=(VPCr+Qb){A.exp[(ΔGATP_ee−C)RT]−1}{A.exp[(ΔGATP_ee−C)RT]+B}

where A = 1 in a simple substrate-to-product reaction [123], B relates to thermodynamic reversibility, C is the value of ΔG_ATP_ at which flux is zero and which has a physiological interpretation [112], and RT ≈ 2.57 kJ/mol. Please note that the calculations based on these three different models must not, in general, provide similar numerical values of Q_max_, and, therefore, are not directly comparable.

It is worth mentioning that the PCr dynamics during the actual exercise can also be used for ATP synthesis rate calculation. However, as the exercise must not always be purely aerobic [63], glycogenolytic ATP synthesis also must be considered. The calculation is, therefore, not as straightforward as in the case of using recovery data. The relevant methods for the quantification of oxidative and glycogenolytic ATP syntheses from ^31^P-MRS data acquired during different types of exercise have been reviewed elsewhere [3].

#### 2.2.2 Hardware requirements

The metabolic processes under investigation are relatively fast, with τ_PCr_ on the order of tens of seconds in healthy individuals. Thus, for the measurement of the dynamics of ^31^P metabolites, the exercise must be synchronized with the MR data acquisition and performed on an MR-compatible device inside the MR system. The design of the device depends on the complexity of tasks it has to fulfill. In particular, if the only goal is to cause appropriate perturbation in ^31^P signals, e.g., at least 15% depletion in the PCr signal, very straightforward tactics can be used. The simplest include the use of rubber resistance bands for plantar flexion exercise of the calf muscle [124], [125], or the recently proposed use of a dual-pocket bag filled with weights and strapped to the ankle of the participant for knee extension exercise of the quadriceps [126]. Although these approaches are very simple and easily applicable in clinical studies [126], they lack the ability to measure additional parameters, e.g., generated force or total energy output, which are commonly provided by the more complex MR-ergometers.

There is an increasing amount of commercial MR-ergometers for different exercise types available [127], [128], [129], [130], with design and controlling software that enable exact workload calibration and recording of force, work, and trajectory or flexion angle. However, due to their costs and/or specific application demands, there are many more built-in-the-lab devices used worldwide [20], [104], [131], [132], [133], [134], [135], [136], [137], [138], [139]. Basic designs apply mechanical or pneumatic workload settings. The first is based on a system of pulleys that transfers the workload outside the magnet bore or even outside the magnet room with a set of counterweights or variable filling of the water-tank at the end of the pulley. A pneumatic design uses pistons pressured by compressed air for workload adjustment. Whether buying a commercial ergometer or constructing one, the experimental design must be considered, i.e. metabolism of which muscle group is to be investigated and what type of exercise is suitable for the task at hand. This is of particular importance when a multi-centric study is planned, and the results from dynamic experiments performed at different research sites are to be directly compared [20].

#### 2.2.3 How to choose the right exercise protocol?

The choice of exercise protocol might have a significant influence on the muscle ATP turnover measured; however, there is a lack of systematic studies [6]. Moreover, it is important to mention that there is no optimal exercise protocol for every application and the choice effectively depends on the equipment and research question at hand. The four basic types of exercise that differ in the ATP synthesis pathways during exercise [63] are (i) ischemic exercise, (ii) pure aerobic exercise under steady-state conditions or (iii) during work jumps, and (iv) mixed exercise. As the blood/oxygen supply is cut off during ischemic exercise, oxidative ATP synthesis is negligible, and therefore, the only source of ATP is the glycolysis. On the other hand, during pure aerobic exercise, the contribution of glycogenolysis can be neglected and the oxidative pathway is solely responsible for ATP production. In mixed exercise, both pathways (oxidative and glycogenolytic) contribute to total ATP production. The two basic exercise modes are isometric, when the muscle tenses without changing its length, and isotonic, which can be further defined either as shortening or as lengthening. The selected design of the exercise, i.e. single workload or incremental, the length of exercise, and the duty cycle influence whether and when the steady-state condition, i.e., the absence of net changes in metabolite concentrations, will be met. A steady-state condition s typically achieved approximately 2 min after the onset of the exercise with a reasonable duty cycle (≈once every 2 s), at a stable moderate workload (20%–40% of maximal voluntary contraction force [MVC]) [140], [141], [142]. If only the oxidative ATP production is of interest, and PCr recovery data are to be used, basically any exercise mode can be selected without any major influence on measured Q_max_ [6]. A special case is a protocol, in which the muscles are directly electrically stimulated rather than performing exercise, and in which a tendency toward lower Q_max_ values has been observed [6].

Then again, this does not mean that careful planning of the exercise protocol is not necessary. One of the main points to be considered is the workload, particularly whether a fixed-value or a proportional workload should be used. The use of a fixed workload for each subject is typical when the differences between individual subjects are of interest, or when no large differences between subjects of the same metabolic group are expected. Although simple and not requiring additional complex setup for calibration measurements [128], [134], [143], such uncalibrated dynamic ^31^P-MRS experiments can lead to increased variability in the PCr depletion and/or a pH drop among subjects [20]. Normalization of the workload to a fraction of MVC substantially reduces the experimental variability. Pre-experimental measurement of MVC should be performed in an exercise setting identical to that in the dynamic experiment [126], [137], or at least should involve the same muscle groups [143]. Consistency of the protocol is also of high importance, as every subject should undergo the exact same examination. This might go without saying for each individual study, but must be also considered for follow-up and/or if a multi-center study is planned [20]. Furthermore, if repetition of more exercise-recovery bouts is planned within a single examination, there must be enough time reserved for physiological recovery of the muscle. It has been shown that results of a second exercise can be influenced, particularly in the presence of strong acidification [144], if the muscles did not have enough time for sufficient recovery. To prevent this influence, a minimum of 15 min of rest is suggested between two bouts of high-intensity exercise [145].

#### 2.2.4 Exercise-induced changes in ^31^P-MR spectra

The most prominent exercise-induced alteration in ^31^P-MR spectra is the change in signal amplitudes of PCr and Pi. However, this is not the only effect on the ^31^P-MR spectrum.

The impact on resonance frequency of Pi has been implied, but not discussed. The chemical shift of Pi is strongly pH-dependent, and the intra-myocellular pH changes during exercise, i.e. rises at the onset of exercise due to the CK reaction and then decreases to a new level depending on the exercise intensity. Thus, the Pi signal dynamically changes its position in the spectrum during muscle exercise (Fig. 4a and 4c). If at any point during the exercise two or more sources with different pH contribute to the acquired signal, more than one Pi signal can be identified in the spectrum [119], [137], [146], [147], [148]. A potential source of Pi splitting could be a compartmentation of pH within one muscle group due to muscle fiber heterogeneity [146]. This hypothesis can be supported by the observation of carnosine peak-splitting by localized ^1^H-MRS of skeletal muscle [149] after exhausting exercise. However, recent studies at ultra-high field strength (i.e. 7 T) found no Pi-splitting when the signal was acquired from a single muscle group and attributed the Pi-splitting to the acquisition of the combined signals of several muscles with different pH [137], [150]. In any case, if Pi-splitting is observed and more than one pH value is calculated, the total Pi equals the sum of all Pi signals, and overall pH can be calculated as a weighted mean of the individual [119].

Another exercise-induced effect is the alteration of signal linewidths. First described at the Pi resonance line, it was also recently detected for the PCr resonance [137]. In general, during exercise, the linewidth of Pi, and, to a lower extent, also of PCr, increases and during recovery slowly returns to its original values. Unlike in Pi, where the line-broadening occurs mainly due to alterations in intramyocellular pH and was attributed to heterogeneous muscle compartmentation [146], the potential cause of PCr line-broadening is the deoxygenation of myoglobin during exercise and its over-oxygenation during recovery [137], similar to the blood oxygenation level dependent effect well known from functional MR imaging [151].

### 2.3 ^31^P-MRS saturation transfer

Another ^31^P technique that provides an insight into the reaction kinetics of energy metabolism is called magnetization transfer (MT). Its most commonly used form is saturation transfer (ST). ST uses the transfer of magnetization between nuclei that are linked by chemical exchange [3]. This technique allows non-invasive measurement of unidirectional exchange rates and metabolic fluxes under steady-state conditions. Thus, ST does not require additional exercise equipment, like dynamic ^31^P-MRS, and it is also applicable in organs that cannot be directly challenged by exercise, e.g., brain [5], [152], [153], liver [17], [19], [154] or heart [152], [155], [156], and potentially also in weak or injured skeletal muscles. On the other hand, the physiological interpretation of the measured data is not as straightforward, and, as such, does not have the same meaning as in dynamic ^31^P-MRS [157], [158], [159].

#### 2.3.1 Underlying principle

**Fig. 5:**
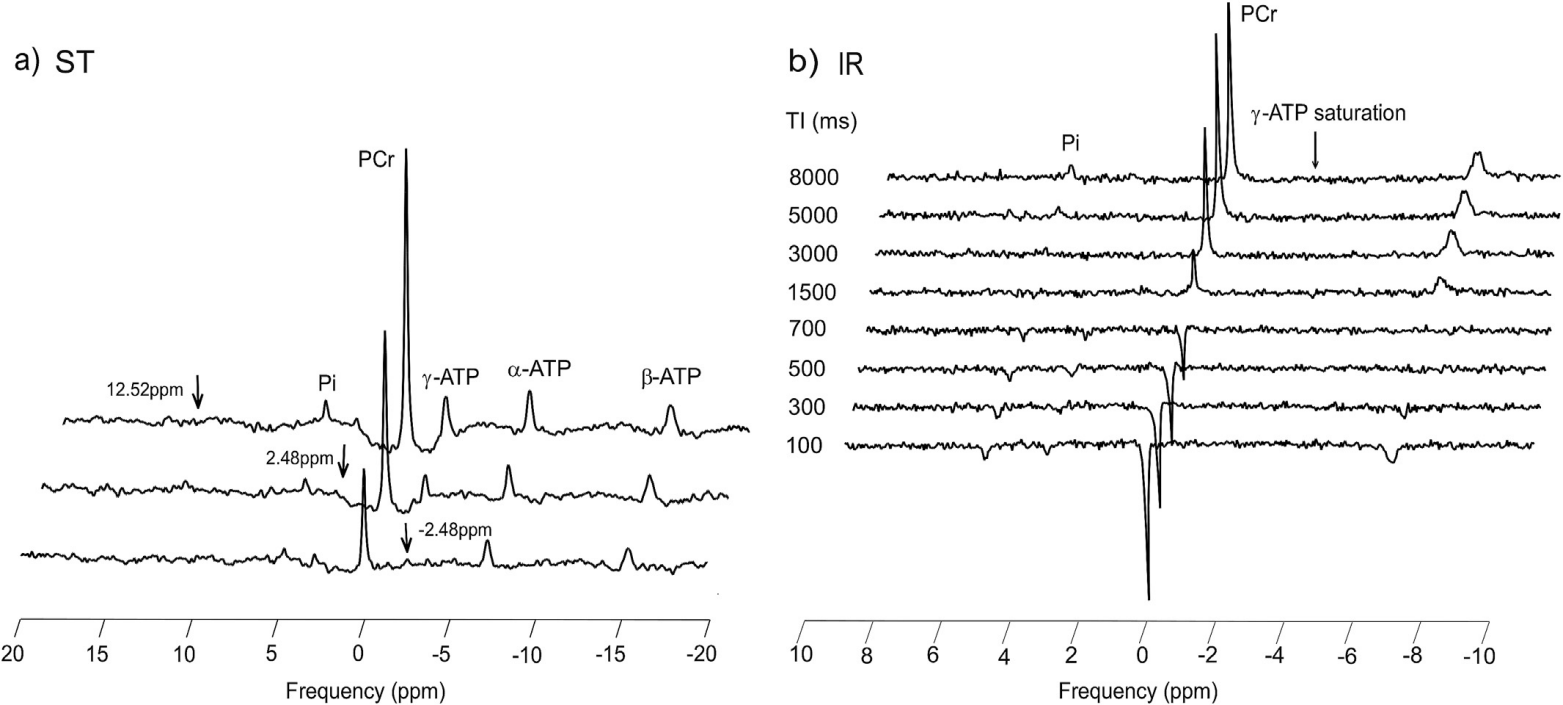
Standard continuous saturation transfer (cST) experiment performed in the gastrocnemius medialis muscle at 7 T. Saturation of the γ-ATP resonance and control spectra are depicted in a) and the inversion recovery (IR) experiment with continuous saturation of γ-ATP to determine the apparent T_1_^app^ is shown in panel b). Arrows depict the saturation frequency in each experiment, i.e. in a) saturation of γ-ATP at −2.48 ppm (bottom); control saturation for the PCr-to-ATP reaction at 2.48 ppm (middle); and control saturation for the Pi-to-ATP reaction at 12.52 ppm (top). Figure was adapted from Ref. [142].

The rate of chemical reaction between two metabolites can be studied by ^31^P-MR through selective irradiation of one metabolite involved in the reaction in order to perturb its magnetization from the equilibrium state, and then, measuring the effect on its exchange partner [160], [161]. One of the most-studied reactions by ST is the creatine-kinase (CK) reaction, described earlier by equation (6). In this reaction, CK catalyzes the exchange of the last phosphate moiety of the ATP molecule (γ-position) to PCr and back. In the standard ST experiment, the γ-ATP resonance frequency is selectively saturated, which results in a reduction in the PCr signal, as depicted in Fig. 5a, due to the forward flux in the CK reaction, the unavailability of unsaturated phosphate to replenish PCr via the reverse reaction, and the fact that the exchange rate is sufficiently faster than the intrinsic T_1_ (T_1_^int^), i.e. T_1_ that would be measured in the absence of any chemical exchange [158]. The fractional reduction in the PCr signal from its equilibrium (M_0_) to the steady-state value (M_z_) is then equal to the pseudo-first-order forward rate constant (k), taking into account the apparent T_1_ of PCr (T_1_^app^), measured with the γ-ATP signal saturated [152]:

(15) k=(1−MzM0)T1app

The T_1_^int^, if of interest, can be calculated using k and T_1_^app^ as follows:

(16) 1T1int=1T1app−k

The pseudo, or apparent, designation of the first-order rate constant *k* is used because the studied reaction is enzymatic, and therefore, the rate constant calculated from the ST experiment is a complex function of the reactants and products, and, as such, does not have the direct meaning as in a non-enzymatic reaction [158]. The forward metabolic flux (F) is given by the product of *k* and the concentration:

(17) F=k.[PCr]

Selective saturation of the γ-ATP resonance also affects the Pi resonance (Fig. 5a) through the ATP hydrolysis, as described in equation (5), and its reverse, i.e. the ATPase reaction. Therefore, the unidirectional rate and metabolic flux of ATP production can also be measured via ^31^P ST, by means of equations (15), (17), using the magnetizations, T_1_^app^ and the concentration of Pi.

#### 2.3.2 ^31^P saturation transfer techniques

As stated above, to calculate unidirectional rate constants, the knowledge of three parameters (M_z_, M_0_, and T_1_^app^) is necessary. Based on the approach how these parameters are measured, we can differentiate several ST techniques. Standard ST uses low-power continuous irradiation (cST) aimed at the γ-ATP resonance frequency. Currently, due to the limitations of modern whole-body MR systems that do not allow extremely long pulses, a pseudo-continuous irradiation, which is performed by a train of very long saturation pulses with minimal inter-pulse delays, is used instead. On the other hand, pulsed ST (pST) uses a specific γ-ATP saturation scheme, e.g., DANTE [162] or BISTRO [163]. In either case, when the applied irradiation nulls the magnetization of the γ-ATP resonance and a new steady state is reached, the M_z_ of the exchange partner can be measured. The equilibrium magnetization M_0_, in the ideal case, is the magnetization without any γ-ATP saturation applied. However, as the saturation pulses are not ideally frequency-selective, to account for the direct saturation effect on the metabolite of interest, a control experiment with the saturation mirrored around its resonance frequency has been proposed to provide genuine M_0_ [164] (Fig. 5). To measure the T_1_^app^, the standard ST experiment involves a separate inversion recovery (IR) experiment with continuous irradiation of the γ-ATP resonance frequency (Fig. 5b). Finally, equation (15) can be used to calculate k.

Alternatively, a progressive ST (prST) experiment exploits the increasing duration of the selective γ-ATP irradiation. Thus, the acquired magnetization of exchange partners becomes a function of irradiation time (t_ir_) and serves to extract both k and T_1_^app^ [165]:

(18) M(tir)M0=1−kT1app.exp(−tirT1app)

Potential issues with the calculation of k may arise in case of incomplete irradiation of γ-ATP, as both equations (15), (18) ignore the residual magnetization of the “saturated” γ-ATP [166]. In addition, introduction of a control saturation experiment that solved the direct saturation problem potentially carries the issue of radiofrequency spillover. Equations that correct for either incomplete saturation [166], or the radiofrequency spillover [167], i.e. combination of the effects of direct saturation and ongoing chemical exchange during the control experiment, have been proposed. More recently, an exhaustive analysis of the effects has been performed, providing equations that account for both potential issues [168]. As an example, based on these reported corrections, equation (15) can be extended to:

(19) k=(1−MzM0).[M0ATP(M0ATP−MzATP)]T1app

where M_z_ and M_0_ are, as previously defined, steady-state and equilibrium magnetization of the exchange partner (e.g., PCr or Pi), while M_zATP_ is the residual magnetization of γ-ATP at steady-state and M_0ATP_ is the magnetization of γ-ATP measured in the control experiment. These issues deserve particular attention at lower field strengths, with the relatively low spectral dispersion. At ultra-high fields, e.g. 7 T, only a minimal effect can be expected and equation (15) is considered to be quite accurate [154], [168].

Up to this point, we have considered only the two-site ST experiments for the measurement of the rate constants of the forward PCr-to-ATP and Pi-to-ATP reactions. Although both these reactions involve ATP, this simplification of the exchange system can be made, because there is no direct exchange between PCr and Pi [169]. However, if the rate constants of the reverse reactions, i.e. ATP-to-PCr and ATP-to-Pi, are to be measured, a slightly more complicated three-site ST experiment is required. This involves either performing two consecutive saturation experiments, aimed at PCr and Pi resonance frequencies, and two control experiments, correspondingly mirrored around the γ-ATP resonance, or including a measurement with simultaneous saturation of both. A detailed description of the necessary experiments and the related equations for the calculation of *k* can be found in Ref. [169].

Another ^31^P-MR technique that can potentially be used to measure both forward and reverse reaction rates in one experiment is a special case of MT called inversion transfer (IT). An IT experiment does not require long saturation pulses, nor complicated saturation schemes, but rather, uses a single frequency selective inversion pulse targeted at the γ-ATP resonance frequency. The inversion of γ-ATP causes a signal decrease and a consecutive recovery of the exchange partners, i.e. PCr and Pi. Magnetizations of PCr, Pi, and γ-ATP, acquired after different inversion times, are used to calculate both forward and reverse exchange rates. However, there is no simplification of the Bloch equations for the magnetization in a two-sided chemical exchange reaction when using IT [170]. An in vivo comparison study between ^31^P ST and IT in human skeletal muscle and liver at 3 T, which provides all the necessary equations for IT, has been recently reported [19]. The high potential of IT for the measurement of both reaction rates can potentially be hindered by the strong dependence of IT on the efficiency of the frequency-selective inversion [19]. The use of an asymmetric adiabatic inversion pulse, which targets frequencies downfield from PCr, i.e. all ATP resonances, was shown to improve the accuracy of the Pi-to-ATP reaction rate determination at 3 T [171]. To amplify the effects of magnetization exchange for ATP synthesis measurement at 7 T, a band inversion targeting all ATP resonances plus the PCr resonance frequency, was proposed recently [172]. This technique is based on the notion that PCr can temporarily store inverted magnetization and transfer it to γ-ATP [173], and thus magnify the MT between γ-ATP and Pi.

#### 2.3.3 Does an ST experiment have to take so long?

One of the main limitations of a standard ST experiment is that it requires relatively long measurement times. The reason for this is threefold: first, it requires a long TR to allow full relaxation, and the T_1_s of the ^31^P metabolites are characteristically long [174]; second, precise measurement of T_1_^app^ via IR requires several (e.g., eight) inversion times; and third, to reach sufficient SNR, extensive signal averaging is required, particularly in the case of the Pi-to-ATP reaction. The SNR increase at ultra-high fields (i.e. 7 T) allows for significantly shorter examination times for ST experiments [39], [153], [154]. Alternatively, several rapid ST techniques [155], [156], [175], [176], [177] have been proposed to increase the temporal resolution of the ST measurement. All of these techniques are based on the acquisition of partially T_1_-saturated spectra, through the utilization of short TRs and on the reduction of total scans needed for the calculation of *k*. Here, we provide an overview of these techniques; more details can be found in the corresponding papers.

Timing optimization of the pre-saturation delays and a BISTRO saturation train length, based on rapid measurement of T_1_^int^ [178] and confident estimate of *k* range, based on reported literature values and/or prior experience, is one of the ways to achieve a significant reduction in the time required for a prST experiment [175]. Another rapid ST technique is called the four-angle saturation transfer (FAST) technique [155]. As the term already implies, this technique is based on the acquisition of only four spectra, two sets of γ-ATP saturated and control spectra, with optimized flip-angles (FAs), under partially saturated conditions (short TR), using the dual-angle method [179] to calculate the T_1_^app^. This technique has also its FASTer and FASTest versions for repeated experiments, when one or both of the low flip-angle acquisitions are omitted, making certain assumptions about the experiment. An exact knowledge of the FAs in the whole excited volume is crucial for the FAST technique. Thus, adiabatic excitation [155], or spatial localization with known FA distribution are applied [142], [180]. A similar approach to the FASTest technique requiring two acquisitions with arbitrary TRs and FAs is called the T_1_ nominal (T_1_^nom^) method [176]. An alternative approach that applies variable TRs rather than alternating FAs is the triple repetition time saturation transfer (TRiST) method [156]. The two-TR method [181] is employed to calculate the T_1_^app^ from two acquisitions with saturated γ-ATP with long and short TR and the third acquisition is the control experiment that provides *M*_*0*_. A recently reported, modified version of TRiST that is based on prior knowledge of intrinsic T_1_ (T_1_^int^) and requires only two acquisitions, i.e. fully relaxed M_z_ and M_0_, is the so called two-repetition time ST (TwiST) method [177].

#### 2.3.4 Interpretation of ST measurements

As the ST experiment does not yield a net chemical flux, the physiological interpretation of the ST results in skeletal muscle is not straightforward. It has been clearly demonstrated in many recent literature reviews that the unidirectional Pi-to-ATP metabolic fluxes measured by ^31^P ST techniques in skeletal muscle at rest significantly overestimate the oxidative ATP synthesis, and therefore, differences in ST results cannot be directly interpreted as alterations of mitochondrial function or capacity [157], [158], [159]. The main reasons are that the overall unidirectional Pi-to-ATP flux contains a major glycolytic component and both turnover reactions operate close to equilibrium, i.e. the net rates of both glycolytic and oxidative ATP synthesis are low at rest [158], although the influence of chemical exchanges with smaller metabolite pools or enzyme-bound metabolites also cannot be fully excluded [157]. Still, the ST measurement of the Pi-to-ATP reaction provides a valid tool for the non-invasive examination of tissue energy metabolism, as it tends to change in pathology, e.g., in insulin resistance, in the same direction as other metabolic measures [182]. The interpretation of the measured PCr-to-ATP reaction flux is much more direct, as it is reasonable to equate the ST measured flux with the overall flux through the CK reaction [159].

To obtain a reliable measure of mitochondrial ATP turnover using ^31^P-MR ST experiment, glycolytic synthesis has to account for just a fraction of the total measured flux, as is the case in working muscle [4], [159]. Initial attempts to measure the Pi-to-ATP flux in human skeletal muscle during steady-state exercise have been published simultaneously only recently [141], [142]. One study utilized an extended protocol consisting of two exercise periods, including saturation, control, and no-saturation measurement in the first period, and IR in the second period [141]. The other study used the FAST technique to measure the Pi-to-ATP flux, and thus, the application of a shorter exercise protocol was sufficient [142]. Both studies reported an increase in the measured flux in exercising muscle compared to rest, as expected, due to the increased ATP demand met by oxidative ATP synthesis. This opens new opportunities for studies of human muscle metabolism by ^31^P-MRS.

### 2.4 Interrelation between static, dynamic, and ST ^31^P-MRS

In the previous chapters, we have described three approaches for the non-invasive measurement of muscle energy metabolism using ^31^P-MR. Although each of them extracts different information and provides different measures, their focus is fairly similar, and therefore, some interrelations can be expected. In the next paragraphs, we will describe some of these, as reported in the literature.

First of all, mitochondrial capacity estimated from the dynamic ^31^P-MRS experiments was found to relate to the recently observed, resting alkaline Pi signal (Pi_2_), which was assigned to originate from the mitochondria [92], [95]. A good agreement between the measured relation between Pi_2_/Pi and the recovery time constant, τ_PCr_, and a model prediction was also found [95]. In addition, there was also a positive correlation reported between [Pi_2_] and Q_max_, as well as Pi_2_/Pi and Q_max_ [92]. The same study [92] also found a negative correlation between Q_max_ and the [PDE], in particular the [GPC], measured at rest. The link between GPC and energy metabolism is not entirely clear, but simultaneous observations of impaired oxidative metabolism and elevated PDE levels are not that rare [66], [67], [68], [69], [183]. The Pi-to-ATP metabolic flux measured by ST at rest has also been correlated with Pi_2_/Pi [92]. Similarly, a negative correlation between [PDE] and Pi-to-ATP flux measured at rest has been reported in the literature [50], [92]. Recently, a relationship has been described between the [PME] and Pi-to-ATP metabolic flux assessed by ^31^P-MRS ST, when measured at rest as well as during steady state exercise [141]. All these relations suggest that high-quality ^31^P-MR spectra acquired at rest can provide surrogate markers of skeletal muscle energy metabolism.

Several studies have also investigated the potential relations between the parameters of dynamic examinations and ST measurements. The relation between the unidirectional Pi-to-ATP flux and the PCr time recovery constant, τ_PCr_, is of particular interest, as τ_PCr_ is often used as an indicator of mitochondrial metabolism. While a report of a negative correlation between them can be found [184], other studies did not observe this [141], [143], [185]. On the other hand, a positive correlation between the Pi-to-ATP flux and Q_max_ has been found across volunteers with various training status [92], [143], [184]. A correlation between the initial recovery rate (V_PCr_) and the Pi-to-ATP flux measured during steady-state muscle exercise, as well as between V_PCr_ and the increment in the Pi-to-ATP flux from rest to exercise value, has also been reported [141]. We can summarize that, even though ST measured at rest does not provide a direct measure of oxidative metabolism, it provides a relevant parameter of energy metabolism that correlates with mitochondrial capacity, and can, therefore, be used as an alternative when dynamic examination is not possible.

### 2.5 Is the metabolism of every muscle the same?

As skeletal muscles differ in muscle fiber composition, their metabolic activity is also not completely alike. Hence, measured metabolic values are unique for each muscle/muscle group and are not directly transferable to another one. When making conclusions based on the ^31^P-MRS data, it is, therefore, important to know the anatomical source of the measured signal. The use of sensitive surface coils and simple pulse-acquire sequences in ^31^P-MRS examinations provides a coarse localization of the measured signal restricted by the sensitive volume of the coil. However, when the sensitive volume covers more muscle groups with a different metabolism and/or recruitment in the exercise challenge an additional localization technique should be applied. A typical example of when such signal localization is necessary is plantar flexion exercise, as the involvement of the gastrocnemius and soleus muscles is different [147], [186], [187], [188], [189], and therefore, the acquisition of a combined signal can bias the results [137], [150] and affect the comparability of results between research sites [20]. Localization strategies for static and dynamic ^31^P-MRS are discussed later in this review.

## 3 ^31^P-MRS of human liver

The liver is the largest organ within the human body and its superficial anatomical position makes it suitable for in vivo MRS experiments using surface coils. The liver is responsible for the metabolism of carbohydrates, lipids, and circulating proteins, and for detoxification of the body's waste products. It is the most important site for the metabolism of drugs and alcohol. Bile is produced in the liver, which is important for the digestion of fats in the gut, and also acts as a transport medium for the excretion of bilirubin and certain drugs. Since the indirect clinical and laboratory measures of hepatic function may be subject to extrahepatic influences, a direct non-invasive measure is of great importance.

^31^P MRS provides information about human liver metabolism in a non-invasive manner [17], [57], [190], [191], [192], [193], [194], [195], [196], [197], [198], [199], [200], [201], [202], [203], [204], [205]. As mentioned in the paragraph about the information content of the ^31^P-MR spectrum, the main feature of the healthy liver ^31^P spectrum is the lack of PCr signal, due to the fact that, under normal conditions, hepatocytes do not express creatine kinase. Alterations in hepatic energy metabolism are typical for inflammatory and neoplastic liver diseases. During the past few decades, the metabolic state of the resting liver has been studied in a wide range of pathologies [206]. These included viral [199], [207], [208] and alcoholic liver disease [191], [209], cirrhosis [195], [196], [197], [210], [211], [212], [213], non-alcoholic fatty liver [201], [205], as well as insulin resistance, and/or type 2 diabetes [202], [214]. Moreover, changes in the ^31^P-MRS pattern were associated with liver metastases [190], [192], [215], [216].

### 3.1 ^31^P-MRS of the liver at rest

**Fig. 6:**
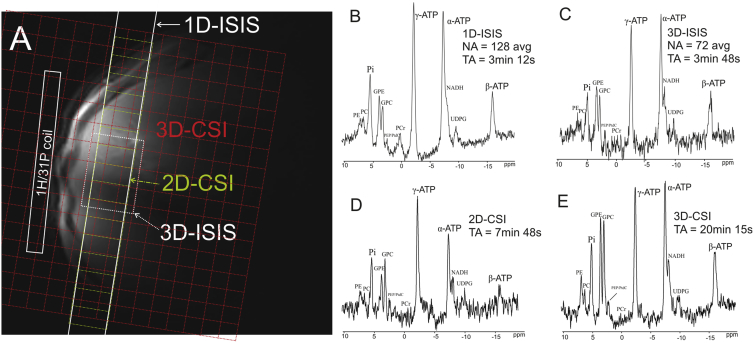
Typical ^31^P-MRS localization strategies for liver examinations. Localizer image of the human liver with indicated surface coil position overlaid with different localization volumes is given in a). Slab-selective 1D image-selected in vivo spectroscopy (ISIS) localization with the slab parallel to the coil, as shown by full white lines provides the spectrum depicted in b). The spectrum in c) was acquired by a single voxel 3D-ISIS technique visualized by the white dotted rectangle. 2D chemical shift imaging (CSI) localization, shown as the yellow dashed matrix, delivers the spectrum depicted in d). The spectrum in e) was acquired using a 3D-CSI technique – red dotted matrix. Figure was reproduced from Ref. [275].

To avoid possible contamination from muscle tissue, gall bladder, or adjacent liver tissue, localization of the hepatic ^31^P-MRS signal must be achieved [8], [17], [53], [217]. To this end, a variety of different strategies (Fig. 6) has been proposed and implemented [22], [37], [218], [219], [220]. These localization techniques are discussed in more detail in the next chapter. The full list of possible clinical applications and findings of hepatic ^31^P-MRS is out of the scope of this methodological review and can be found elsewhere [203], [221], [222], [223]. Here we provide only a short list of consensus findings.

#### 3.1.1 Hepatic energy homeostasis (ATP)

Hepatic energy homeostasis can be assessed through the measurement of ATP levels. This has been extensively used in ^31^P-MRS studies focused on investigating the viability of stored and transplanted livers [224], [225], [226], [227]. Although liver transplantation has proven to be a safe and effective means of treatment for end-stage organ failure, the ischemic damage sustained during cold storage can potentially affect the transplant viability. The amount of ATP present in the stored liver tissue reflects the length of cold storage [223]. If the measurement of hepatic energy homeostasis can predict response and recovery from partial hepatectomy, patient selection for surgical intervention could be improved. Another potential clinical application of ^31^P-MRS is the identification of prospective transplant rejection. The current gold standard is histology from a percutaneous liver biopsy. However, since changes in rejection may be patchy, biopsies may be subject to sampling errors. Moreover, biopsy itself is not without risk of morbidity or mortality.

ATP levels, measured by ^31^P-MRS, are also altered in patients with diffuse liver diseases, or general systemic metabolic diseases that also affect the liver, e.g., obesity or type 2 diabetes. Examples include non-alcoholic fatty liver disease (NAFLD), as well as non-alcoholic steatohepatitis (NASH) [228]. Obese subjects [229] and type 2 diabetic patients [230] were also shown to have more depleted baseline ATP reserves.

#### 3.1.2 Alteration of cell membrane metabolism (PMEs and PDEs)

Whenever the cell membrane metabolism is altered, ^31^P-MRS may identify such changes as the, in the ^31^P-MR spectrum present, PMEs are cell membrane precursors and PDEs are cell membrane degradation products. PME/PDE impairment was, for example, reported in patients with liver cirrhosis [195], [196], [197], [210], [211], [212], [213].

Neoplastic changes are also associated with cell membrane reorganization, and, therefore, the most obvious and most consistent abnormality in spectra from liver tumors and metastases is an elevation in the PME region, which may be considered a diagnostic discriminator [231]. Subsequent MRS studies after treatment showed a reduction in PME levels, and, finally, after a successful therapy, the spectra became similar to those of healthy subjects. The majority of studies have involved easily accessible tumors; for example, lymph node metastases, bone tumors, lymphomas, and soft tissue tumors [231], [232], [233]. The findings of these studies suggest that falling of initially increased PME levels indicates that the drugs are reaching the target cells and affecting tumor cell metabolism, which may be of considerable clinical importance.

The ^31^P MR spectra from isolated human livers in cold storage, and those from large animals preserved under the same conditions, contain resonances attributable to PME and PDE, which diminish with time. However, the prediction of primary graft dysfunction using PMEs, was shown to be unreliable at the clinical field strength of 1.5 T [234].

#### 3.1.3 The metabolically challenged liver

Another exciting line of research involves studies of hepatic metabolism after metabolic perturbations by exogenous substances. For example, type 2 diabetes mellitus is characterized by fasting hyperglycemia and an excessive, prolonged rise in plasma glucose concentrations after glucose or meal ingestion. The infusion of L-alanine or fructose bolus induces rapid and consistent changes of ^31^P MR spectra [193] and may serve as a tool for studies of gluconeogenesis [235], [236], [237], [238], [239]. Injections of acetaminophen, i.e. self-poisoning, were shown to cause a decrease in the concentration of all phosphorus metabolites in parallel to a decline in the synthetic ability of the liver. In severe cases, ATP and PDE levels fell to ∼20% of their normal concentrations [240].

#### 3.1.4 Other potential biomarkers

Recent studies have pointed out the potential clinical importance of ^31^P metabolites other than ATP, PDE, and PME signals. In particular, NADH – a marker of inflammation and fibrinogenic activity – is increased in patients with NASH and in those with cirrhosis, but it is not increased in patients with NAFLD [201]. Increased fructose intake over a four week period was also shown to cause an increase in NADH in healthy volunteers [40]. Moreover, PtdC – a dominant component of the human bile MRS signal – contribution to the hepatic in vivo ^31^P-MRS signal was identified [8] and independently confirmed [217]. Further studies should investigate the potential use of the PtdC resonance for metabolic studies of the liver, gallbladder, and bile ducts. Changes in biliary PtdC may be an indicator of malignancy and dynamic metabolic perturbation, representing cell breakdown, death, and cellular regeneration [241]. The defects in biliary PtdC secretion may play a key role in the pathogenesis of bile duct and liver diseases [241], [242], [243], [244].

#### 3.1.5 Saturation transfer in the human liver – the ATP synthase flux

The principle of the ST technique, as well as all the methods presented for skeletal muscle, can be applied to the human liver as well. There are, however, a few differences. As there is no PCr present in healthy liver tissue, the application of ST ^31^P-MRS for the non-invasive measurement of chemical exchange rates in the human liver is limited to the assessment of flux through the ATPase reaction. This does not diminish the value of ST, as the estimation of the hepatic Pi-to-ATP exchange rate provides important information about liver physiology. As the Pi-to-ATP exchange rate in skeletal muscle differs from the hepatic rate [19], a dedicated localization technique has to be used to avoid signal contamination from abdominal muscles. Further, due to the increased distance from the commonly used surface coil and the relatively low metabolite concentrations in liver, the full ST examination in human liver requires extensive averaging. Thus, the required measurement times reported for clinical MR systems (i.e. 3 T) are up to 1.5–2 h [17], [19]. The examination time can be significantly shortened, i.e. to ∼25 min, with relatively high repeatability, if the hepatic ^31^P-MRS ST measurements are performed at 7 T [154], [245]. This opens the possibility to include the estimation of the Pi-to-ATP exchange rate by ^31^P-MRS ST in clinical studies of the human liver in vivo [245]. Initial patient studies have reported a decreased hepatic Pi-to-ATP metabolic flux, caused by reduced Pi concentrations, in type II diabetic patients compared to matched healthy controls [202], as well as lower Pi-to-ATP exchange rate constant in patients with nonalcoholic steatohepatitis compared to healthy controls and patients with simple steatosis [205], [245].

## 4 Localization of ^31^P-MR signal for investigations of skeletal muscle and liver

^31^P-MRS is commonly, but not exclusively [22], [124], [246], employed with the use of highly sensitive surface coils. Nevertheless, signal localization by restricted sensitivity volume of these RF-probes does not allow a distinction between the signals that originate from different anatomic and/or morphologic compartments. Thus, particularly for liver ^31^P-MRS, at least a simple localization strategy must be applied. The following paragraphs describe the typical localization techniques (Fig. 6) for ^31^P-MRS of human liver and/or skeletal muscle, but these are also applicable to other organs of interest, e.g., heart or brain.

In general, the T_2_ relaxation times of ^31^P metabolites are relatively short, and thus, so-called “pulse-acquire” or “non-echo” FID-based MRS techniques are generally preferred [15]. Another important parameter to consider is the relatively large spectral dispersion of the ^31^P-MR spectra. Thus, chemical shift displacement error (CSDE) could lead to significant bias in the localization accuracy of ^31^P spectra. The use of CSDE-insensitive methods, i.e. implementation of selective, refocusing, and inversion pulses with relatively large bandwidths, is, therefore, essential [37].

### 4.1 Outer volume suppression

Suppression of the signal from the volume in the vicinity of the volume of interest, e.g., suppression of abdominal muscle signal during MRS of the liver, can be used as a standalone “negative” localization technique, but can also accompany and expand any other localization sequence.

One such technique is based on the utilization of broadband outer-volume saturation (OVS) bands, consisting of spatially selective RF-pulses, inserted into the main pulse sequence. OVS can be effectively used to selectively saturate, i.e. suppress, the ^31^P-MR signal originating from muscles of the abdominal wall, leaving only negligible contamination and also allowing hepatic ST measurements [19]. Then again, this suppression technique is rather SAR intensive, and therefore, would not be applicable at ultra-high fields, i.e. 7 T, particularly, if combined with the saturation pulses for ST experiment.

An alternative option to suppress the superficially originating signals without any additional RF power deposition is to use a surface-spoiling crusher coil [247], [248], [249]. The underlying idea is that a direct current (DC) applied to a crusher coil produces a region of inhomogeneous magnetic field in its vicinity. This effectively means that if the crusher coil is placed over the abdominal wall muscles, the use of DC pulses between RF excitation and signal acquisition can lead to signal dephasing, i.e. spoiling of the transverse magnetization, in the superficially located muscles, leaving the signal originating from the liver intact. The depth of dephasing is proportional to the applied DC current, and the sharpness of the transition between spoiled and unspoiled regions depends on the crusher coil design and placement. Besides, the SAR reduction provided by the crusher coil allows for more SAR intensive sequence design, even at ultra-high fields [248].

### 4.2 Single voxel localization

There is a variety of single-voxel spectroscopy (SVS) sequences available for accurate spatial selection of a cuboid 3D volume, i.e. a voxel, which can be used for single muscle or liver localization. To minimize the influence of T_2_ relaxation and J modulation on the acquired signals, FID-based sequences with a negligible acquisition delay are generally preferred for ^31^P-MRS. The only FID-based SVS technique is the so-called image-selected in vivo spectroscopy (ISIS). It combines eight FID acquisitions with different configurations of spatially selective inversion pulses, preceding excitation, for accurate selection of a 3D voxel [219]. To avoid contamination due to T_1_ smearing under partially saturated conditions, an extended ISIS scheme [250], combining 38 acquisitions for volume selection, is required [37]. This is not a limitation for the measurements of human liver or skeletal muscle at rest, and thus, high-quality spectra, with accurate spatial selectivity of a 3D volume, can be acquired using the ISIS sequence within clinically acceptable measurement times [37]. Nevertheless, the multi-acquisition nature of full 3D-ISIS volume selection makes it prone to movement artifacts and its relatively long acquisition time is impractical for dynamic ^31^P-MRS. Single-shot SVS localization sequences that allow full signal localization in only one acquisition are, therefore, preferred for this purpose. A stimulated echo acquisition mode (STEAM) sequence has been shown to be sufficient for dynamic single-muscle localization at 3 T [133]. However, the temporal resolution of STEAM localization for dynamic examinations is lower in comparison to pulse-acquire sequences, as signal averaging is necessary to retain sufficient SNR for reliable quantification of the measured spectra. The use of ultra-high fields, i.e. 7 T, significantly improves the SNR of the STEAM sequence, allowing higher temporal resolution. However, the CSDE that is caused by the low bandwidth of the spatially selective RF pulses used in STEAM considerably influences the localization accuracy at 7 T. To reduce CSDE, high-bandwidth RF pulses have to be applied for spatial selection [37]. For dynamic ^31^P-MRS, it has been demonstrated that using conventional slice-selective excitation, combined with localization by adiabatic selective refocusing (semi-LASER), can provide high selection efficiency and low outer volume contamination at 7 T [251]. Effective adiabatic refocusing used in semi-LASER has also the potential to yield double SNR compared to STEAM. However, long refocusing pulses within a semi-LASER sequence imply relatively long TE, thus reducing the sensitivity of the sequence for metabolites with short T_2_ or spins undergoing J-modulation, e.g., ATP [251].

### 4.3 Slab-selective localization

Full 3D localization of the volume of interest is often not absolutely necessary; particularly, if surface coils with inherently spatially restrictive sensitivity volumes are used. When the applied pulse sequence (e.g., ISIS) provides localization in only one spatial dimension (1D) and the sensitive volume of the coil is used for localization in the other two dimensions, a so-called slab-selective localization (e.g., 1D-ISIS) is achieved. Such 1D-ISIS localization does not provide spatial information from within the selected volume, which is commonly very large, and therefore, it is ideal for the investigation of homogeneous tissues and systemic diseases. In particular, this type of spatial selection is highly efficient for liver signal localization, as the slab can be positioned parallel to the RF-coil, i.e. avoiding muscle tissue contamination [38]. 1D-ISIS localization has been also shown to provide sufficient volume selection for hepatic ^31^P-MRS ST measurements [17], [154].

**Fig. 7:**
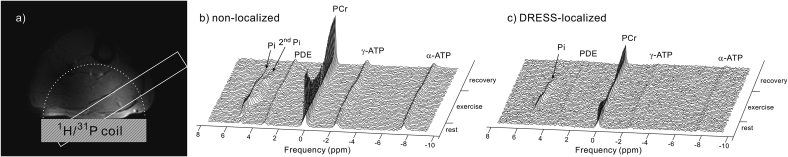
A comparison of spectra acquired in a dynamic examination using simple topical surface-coil localization (non-localized) and a slice-selective, depth-resolved surface coil MRS (DRESS) localization. An in vivo localizer image with the depicted RF-coil position overlaid with localization volumes is given in a). The full line represents the DRESS selection placed over the gastrocnemius medialis, and the dotted line represents the approximate RF-coil sensitivity volume, containing several muscles. Stack plots of the ^31^P spectra acquired during rest, exercise and subsequent recovery are shown for the non-localized (b) and DRESS-localized (c) acquisitions. The spectra are scaled for equal noise to show the lower signal intensity of the localized experiment. On the other hand, the specificity to challenged muscle improves the dynamic range of PCr depletion. Note the Pi split in the non-localized data, which is lacking in the DRESS-localized MRS time course. Figure was reproduced from Ref. [150].

When applied to skeletal muscle, 1D-ISIS can be used for single-muscle localization with FID acquisition and low CSDE, which has been demonstrated for both ST [154] and also for dynamic ^31^P-MRS [252]. However, as 1D-ISIS localization requires a combination of two transients, it might still be prone to motion artifacts during a dynamic examination. A different FID acquisition-based 1D localization sequence, called depth-resolved surface coil MRS (DRESS [253]) using a slice-selective excitation pulse, was presented for dynamic ^31^P-MRS recently [150] (Fig. 7). DRESS has also been successfully combined with frequency-selective saturation pulses to measure muscle-specific unidirectional PCr-to-ATP and Pi-to-ATP fluxes using the standard ST and/or FAST technique [142]. Slab-selective localization has been shown to provide sufficient localization of the superficially located muscles, e.g., the gastrocnemius, for both dynamic and ST ^31^P-MRS, but the localization in only one dimension would not be optimal for deeper muscles.

### 4.4 Multi-voxel MRS localization

The localization techniques for dynamic ^31^P-MRS described above typically target only a single muscle at a time, and therefore, to investigate several muscle groups, repeated examinations have to be performed [187]. Recently, an interleaved semi-LASER acquisition that provides information from two independent voxels in one dynamic experiment, was proposed [254]. However, small, spatially defined injuries, myopathies, or functional deficits in peripheral arterial disease (PAD) can still be overlooked if not present in the acquired volume of interest (VOI). Thus, an option to spatially localize signal from several muscle groups at once would be beneficial. Similarly, ^31^P-MRS investigations of human liver would benefit from spatially resolved acquisitions. MR spectroscopic imaging (MRSI) techniques allow spatially localized mapping of metabolite concentration within a larger VOI, and therefore might provide a suitable option [218], [255].

The basic principle of MRSI can be illustrated through phase-encoding in magnetic resonance imaging (MRI), with the extension of an additional frequency dimension, i.e. the chemical shift dispersion. Therefore, MRSI is commonly referred to as chemical shift imaging (CSI); and we will use these abbreviations interchangeably to accustom the reader to both of them. The key element during any MRSI experiment is a gradient pulse G_n_, which produces a phase shift in the acquired FID, corresponding to the encoded spatial information. Such phase encoding used in all three directions makes ^31^P 3D-CSI completely insensitive to CSDE [53], [256]. ^31^P 3D-CSI was successfully applied in many metabolic studies of human liver [53], [55], [230], [257] or skeletal muscle [94]; however, it can be very time-consuming [258], especially if larger matrix sizes are acquired. A decreased number of encoded spatial dimensions, i.e. 1D or 2D-CSI, could offer multi-voxel acquisition in clinically acceptable measurement times [38], [259]. Unfortunately, standard ^31^P 2D-MRSI sequences suffer from the inherent disadvantages of slice-selective excitation, i.e. CSDE. In addition, time-consuming pulse adjustments and accurate knowledge of FA distribution are mandatory for quantification corrections. To overcome this restraint, a combination of 1D-ISIS slab selection with 2D-CSI spatial encoding has been suggested [220]. This fully adiabatic ^31^P 2D-CSI sequence reduces operator preparation time and the number of correction factors for metabolite quantification.

Although MRSI enables spatially resolved acquisition of ^31^P-MR spectra of the human liver and skeletal muscle at rest, traditional CSI sequences still require relatively long acquisition times, and therefore, do not allow high temporal resolution suitable for dynamic experiments, unless very low spatial resolution [146], specialized protocols [260], and/or rapid encoding strategies [261] are applied. The focus of the next several paragraphs will be only on these specialized cases.

As mentioned, the direct use of ^31^P-MRSI for dynamic examinations allows only low matrix sizes, e.g., 4 × 4, and, even with the implementation of fast Hadamard encoding [262], results in temporal resolution of 96 s [146]. While this allows investigation of the changes in metabolite concentrations and pH from rest to exercise, it is insufficient to reliably map the PCr recovery. To overcome this limitation, a dedicated exercise protocol has been proposed [260], [263]. Repeated contractions followed by short relaxation periods in a gated protocol cause the spectra to reach a new steady state in which the PCr recovery curves of each cycle follow the same exponential as would the PCr recovery after a conventional repetitive exercise. Therefore, each phase-encoding step of the gated MRSI protocol can be acquired during different recovery and the PCr recovery curve can be sampled with high temporal resolution. The spatial resolution of gated MRSI is limited by the need for a repeated contraction-relaxation cycle for each encoding step; e.g., matrix sizes of 8 × 8 voxels require a 16-min exercise protocol [263].

To avoid the need for an extended exercise protocol while acquiring high spatially resolved dynamic data, the slow Cartesian phase-encoding has to be exchanged for a faster trajectory. Recently, a constant-density spiral spectroscopic readout has been proposed for dynamic ^31^P-MRSI experiments [261]. The use of temporal interleaved spirals allowed the acquisition of a 14 × 14 matrix in 10 s; thus, a simple measurement protocol using repetitive exercise can be used. This rapid acquisition readout is traded for spectral bandwidth; thus, the range of resonance frequencies that are covered is rather limited. However, the main metabolites of interest for dynamic experiments, i.e. PCr, Pi and γ-ATP, are within the bandwidth measured with the proposed setup [261].

### 4.5 Selective ^31^P-MR imaging

An alternative approach to MRSI that provides high spatial resolution with reasonable temporal resolution is MRI. However, while in ^1^H MRI the overwhelmingly strong signal of water dominates signals of all other metabolites, there is no such source in ^31^P-MR. Even if PCr was assumed to be the main source of ^31^P-MRI signal at rest, this could change during exercise. In addition, total ^31^P signal does not change during exercise, and therefore, there would be no exercise-induced changes visible in the MR images of the total ^31^P signal. To obtain images that represent only one single metabolite, spectrally selective ^31^P-MRI [264] can be used. The underlying principle of spectrally selective ^31^P-MRI is the use of low bandwidth excitation pulses that selectively excite only the metabolite of interest. Although typically only one metabolite image is acquired [246], [265], [266], [267], simultaneous imaging of multiple ^31^P metabolites, using either interleaved [268], [269], [270] of multi-frequency excitation [271], has also been suggested.

If only one metabolite is to be imaged, the choice for dynamic ^31^P-MRI experiments is clear. Imaging of dynamic changes in the PCr signal has been recently shown to be feasible, with temporal resolution on the order of seconds [124], [125], [267]. High spatial resolution of selective ^31^P-MRI allows good differentiation between multiple muscle groups within the VOI, and therefore, muscle-specific PCr recovery can be evaluated. However, for the evaluation of mitochondrial capacity, dynamic information about other metabolites and, more importantly, about pH, is missing. The simultaneous acquisition of PCr and Pi images with high temporal resolution to obtain additional information about Pi dynamics has been proposed recently [270]. In addition to the observation of Pi dynamics, the phase images were used to calculate the chemical shift of Pi, and thus, also the information about pH can be extracted [270].

^31^P-MRI can also be combined with saturation pulses and used for localized ST experiments [272], [273], [274]. Although the cST approach with an IR experiment for T_1_^app^ measurement has been shown to be feasible at 7 T for the PCr-to-ATP reaction [272], the required measurement time was relatively long (∼60 min). Therefore, to avoid the lengthy IR experiment, the use of a prST experiment was suggested for the PCr-to-ATP reaction rate imaging at 3 T [273], as well as for imaging of the unidirectional Pi-to-ATP metabolic flux at 7 T [274]. Although a selective excitation pulse is used, partial off-resonance excitation of highly concentrated PCr cannot be fully excluded and requires an additional PCr suppression pulse [274].

## 5 Conclusions

^31^P MR spectroscopy and imaging are capable of the non-invasive assessment of human skeletal muscle and liver metabolism. Next to the identification of different pathologies and tissue characterization at basal resting conditions, the dynamic in vivo ^31^P MRS investigation of PCr depletion and resynthesis during ischemia, exercise challenge, and following aerobic recovery, can be applied to monitor ATP handling in skeletal muscle. Dynamic ^31^P MRS has been applied in the field of sports physiology and has increased our knowledge of energy metabolism under challenged conditions. This, again, can be used to phenotype diffuse tissue disease and follow-up different therapy approaches, including pharmacological treatment, nutrition, and life-style intervention. An alternative method of saturation transfer (ST) can monitor the chemical exchange of phosphorous atoms between ATP, PCr, and Pi, and can be used to quantify the energy fluxes in skeletal muscle and liver. Defects of ATP synthase flux in muscle and liver were revealed in type 2 diabetes mellitus and pre-diabetic states. It was also shown that adjusted and accelerated ST measurements can be applied to measure ATP handling in the beating myocardium.

Large frequency dispersion in ^31^P MR spectra allows for simple and coarsely localized skeletal muscle and liver experiments, even at a low field strength of 1.5 T, but the potential of the method can be fully explored at high (3 T) and ultra-high (7 T) field strengths only. Experimental time resolution and/or signal localization prosper from the improved SNR, enabling 2D^31^P CSI of the lower leg in a dynamic exercise-challenged fashion, and clear separation of different muscle groups. Recent methodological developments have focused exactly on the issue of accelerating and refining the measurement without losing substantial physiological information. In the field of hardware, which was not in the scope of this review, RF-probes with increased sensitivity, improved B_1_ homogeneity, and SAR efficacy will further boost this research in the near future. This will further reduce patient examination time and increase the value of ^31^P MRS/I for the biochemical analysis of tissue-specific energy metabolism.
